# Neuroplastic changes induced by long-term *Pingju* training: insights from dynamic brain activity and connectivity

**DOI:** 10.3389/fnins.2024.1477181

**Published:** 2024-09-27

**Authors:** Fangshi Zhao, Linlin Song, Yule Chen, Shaoying Wang, Xiaoyi Wang, Ying Zhai, Jinglei Xu, Zhihui Zhang, Minghuan Lei, Wenjie Cai, Qi An, Dan Zhu, Fengtan Li, Chunyang Wang, Feng Liu

**Affiliations:** ^1^Department of Radiology and Tianjin Key Laboratory of Functional Imaging and Tianjin Institute of Radiology, Tianjin Medical University General Hospital, Tianjin, China; ^2^Department of Ultrasound, Tianjin Medical University General Hospital, Tianjin, China; ^3^Department of Radiology, The Second Hospital of Tianjin Medical University, Tianjin, China; ^4^Department of Radiology, Tianjin Medical University General Hospital Airport Hospital, Tianjin, China; ^5^Department of Scientific Research, Tianjin Medical University General Hospital, Tianjin, China

**Keywords:** *Pingju*, neuroplasticity, dynamic ALFF, dynamic FC, resting-state fMRI

## Abstract

**Background:**

Traditional Chinese opera, such as *Pingju*, requires actors to master sophisticated performance skills and cultural knowledge, potentially influencing brain function. This study aimed to explore the effects of long-term opera training on the dynamic amplitude of low-frequency fluctuation (dALFF) and dynamic functional connectivity (dFC).

**Methods:**

Twenty professional well-trained *Pingju* actors and twenty demographically matched untrained subjects were recruited. Resting-state functional magnetic resonance imaging (fMRI) data were collected to assess dALFF differences in spontaneous regional brain activity between the actors and untrained participants. Brain regions with altered dALFF were selected as the seeds for the subsequent dFC analysis. Statistical comparisons examined differences between groups, while correlation analyses explored the relationships between dALFF and dFC, as well as the associations between these neural measures and the duration of *Pingju* training.

**Results:**

Compared with untrained subjects, professional *Pingju* actors exhibited significantly lower dALFF in the right lingual gyrus. Additionally, actors showed increased dFC between the right lingual gyrus and the bilateral cerebellum, as well as between the right lingual gyrus and the bilateral midbrain/red nucleus/thalamus, compared with untrained subjects. Furthermore, a negative correlation was found between the dALFF in the right lingual gyrus and its dFC, and a significant association was found between dFC in the bilateral midbrain/red nucleus/thalamus and the duration of *Pingju* training.

**Conclusion:**

Long-term engagement in *Pingju* training induces neuroplastic changes, reflected in altered dALFF and dFC. These findings provide evidence for the interaction between artistic training and brain function, highlighting the need for further research into the impact of professional training on cognitive functions.

## Introduction

*Pingju*, also known as Ping Opera, is one of the most popular traditional Chinese operas, particularly prevalent in northern China. It combines a variety of performing arts, including singing, dialogue, acting, and acrobatics, to tell stories that reflect Chinese culture and history (Liu, 2023). The training for *Pingju* begins at a young age, typically around 6–7 years old, and involves rigorous and prolonged practice in vocal techniques, body movements, and emotional expression. Extensive training often leads to significant changes in the brain, a phenomenon known as neuroplasticity, which allows the brain to modify its structure and function in response to new experiences, skills, and environmental changes (Zhang et al., 2018; Zhao et al., 2020; Draganski et al., 2004) and is crucial for learning, memory, and recovery from injury (Wu et al., 2020; Olszewska et al., 2021; Hiraga et al., 2022). For instance, professional musicians and athletes show distinct neural adaptations due to their specialized training (Münte et al., 2002; Nager et al., 2020; Zhang et al., 2021; Gao et al., 2023). The unique combination of skills required in *Pingju* provides an opportunity to study the effects of long-term, specialized training on brain function.

To explore the neural mechanisms related to specialized training, researchers can employ resting-state functional magnetic resonance imaging (rs-fMRI), a non-invasive method that captures spontaneous brain activity by detecting fluctuations in blood oxygen level-dependent (BOLD) signals (Guerra-Carrillo et al., 2014). Previous rs-fMRI studies have consistently shown that specialized training in various professions, such as musicians, athletes, dancers, and seafarers, can result in changes in brain response patterns (Lu et al., 2018; Wang et al., 2018; Vuust et al., 2022; Zhang et al., 2024). Within rs-fMRI, the amplitude of low-frequency fluctuation (ALFF) and functional connectivity (FC) are two key metrics. ALFF quantifies the power of low-frequency oscillations in the BOLD signal, providing a measure of spontaneous local brain activity (Zuo et al., 2010; Liu et al., 2013; Su et al., 2022). On the other hand, FC measures the temporal correlation between BOLD signals from different brain regions, reflecting the functional interactions and synchronization between these regions (Friston, 2011; Liu et al., 2015; Rubia et al., 2019). Together, ALFF and FC offer a comprehensive view of brain function, linking local neural activity with broader interactions between brain regions. This dual approach allows for a deeper understanding of how specific brain regions and FCs contribute to the skills required in *Pingju* performance.

Traditional static ALFF and FC methods provide an averaged view of brain activity over the entire scanning period, which can miss transient yet important neural dynamics. The brain is a highly dynamic system with constantly changing neural activities and interactions, resulting in time-varying patterns of brain activity (Chang and Glover, 2010). Relying solely on averaged brain activity across a time series can, therefore, lead to a loss of valuable insights into underlying neuronal processes. To address this issue, dynamic approaches have been developed to capture the temporal variability of brain activities (Hutchison et al., 2013; Allen et al., 2014; Liu et al., 2017). Dynamic fluctuations in resting-state brain activity have been associated with cognitive and behavioral traits, suggesting that dynamic measures may provide more sensitive biomarkers than static ones in certain contexts. For example, dynamic ALFF (dALFF) captures changes in local brain activity over short time frames, highlighting regional neural dynamics associated with processes like learning and adaptation (Zhu et al., 2024). Similarly, dynamic FC (dFC) monitors shifts in connectivity between brain regions, unveiling changes in network interactions and integration (Preti et al., 2017), with dFC patterns linked to various cognitive functions and adaptive processes (Xu et al., 2022; Li et al., 2023; Matkovic et al., 2023). Thus, employing a dynamic approach enables the exploration of how brain activity adapts and reorganizes in response to specific demands, such as those encountered in rigorous *Pingju* training, offering a more comprehensive understanding of brain function and its plasticity following long-term training.

In this study, we aimed to explore the neuroplastic effects of *Pingju* training by comparing dynamic brain activity and connectivity between professional *Pingju* actors and untrained individuals. Specifically, we utilized dALFF to measure local brain activity and dFC to assess the functional interactions between brain regions. We hypothesized that the intensive, long-term training of professional *Pingju* actors would lead to different patterns of brain activity and connectivity, reflecting the specialized skills and cognitive demands of their profession.

## Materials and methods

### Participants

This study was approved by the Ethics Committee of Tianjin Medical University General Hospital, and written informed consent was obtained from each participant. The study included 20 professional traditional Chinese *Pingju* actors and 20 untrained individuals. The actors were recruited from Tianjin *Pingju* Theater and Tianjin Baipai *Pingju* Theater. Untrained individuals were those without any professional musical education. None of the participants had a history of neurological or psychiatric disorders, nor did their first-degree relatives. All participants, both actors and untrained subjects, were right-handed according to the criteria of the Chinese revised version of the Edinburgh Handedness Inventory (Oldfield, 1971).

### Image acquisition

MRI data were acquired using a 3.0-Tesla MR system (Discovery MR750, General Electric, Milwaukee, WI, USA). The participants were instructed to stay awake with their eyes closed during the scan. Foam pads were used to minimize head motion, and earplugs were used to reduce scanner noise. The rs-fMRI data were obtained using a single-shot echo-planar sequence with the following parameters: repetition time (TR) = 2000 ms, echo time (TE) = 45 ms, flip angle = 90°, slice thickness = 4.0 mm, gap = 0.5 mm, field of view (FOV) = 220 mm × 220 mm, matrix = 64 × 64, number of axial slices = 32, and 180 volumes. High-resolution T1-weighted images were acquired using a brain volume sequence with TR = 8.17 ms, TE = 3.18 ms, inversion time = 450 ms, slice thickness = 1.0 mm, FOV = 256 mm × 256 mm, matrix = 256 × 256, and 188 sagittal slices.

### Image preprocessing

The rs-fMRI data were preprocessed using the Data Processing and Analysis for Brain Imaging (DPABI) toolbox (Yan et al., 2016). Initially, the first 10 time points were removed to allow signal equilibrium. Subsequently, the fMRI data were corrected for temporal differences between slices and head motion. Participants with a maximum displacement greater than 3.0 mm, maximum rotation greater than 3.0°, or mean framewise displacement (FD) greater than 0.3 mm were excluded from further analyses (Power et al., 2012). Following this, individual structural images were co-registered to the corresponding mean functional images, and the transformed structural images were segmented into gray matter, white matter, and cerebrospinal fluid. Using these segmented images, normalization parameters from individual native space to Montreal Neurological Institute (MNI) space were estimated via the Diffeomorphic Anatomical Registration Through Exponentiated Lie Algebra (DARTEL) tool (Ashburner, 2007). The motion-corrected functional imaging data were then normalized to MNI space using these parameters and resampled to 3-mm cubic voxels. Next, spatial smoothing was conducted with an 8 mm full width at half maximum (FWHM) Gaussian kernel. Finally, spurious variances were removed through regression, including linear drift, Friston-24 head motion parameters, and signals from white matter and cerebrospinal fluid (Ma et al., 2022).

### dALFF calculation

The dALFF analysis was conducted using a sliding-window approach based on the Temporal Dynamic Analysis (TDA) toolkit (Yan et al., 2017). Since there is no consensus about the selection of window length, a previous study suggests that the minimum window length should not be less than 1/*f*_*min*_ (the minimum frequency of the time series), as a shorter window may increase the risk of false fluctuations while a longer window could diminish the dynamics (Leonardi and Van De Ville, 2015). Based on this guidance, a window length of 50 TRs (100 seconds) and a step size of 1 TR (2 seconds) was used to characterize dALFF (Fu et al., 2018; Cui et al., 2020). Within each time window, the ALFF map was calculated by applying a fast Fourier transform (FFT) to convert the time series into the frequency domain, decomposing the signal into its constituent frequencies to obtain the power spectrum (Zang et al., 2007). The ALFF value was then computed by taking the square root of the power spectrum within the predefined low-frequency range (0.01–0.08 Hz). The temporal variability of the ALFF (i.e., dALFF) was assessed by calculating the standard deviation (SD) of these maps. For each participant, the dALFF of each voxel was converted into *z*-scores by subtracting the mean and dividing by the SD of the global values.

### dFC analysis

Before dFC analysis, the fMRI data were temporally bandpass-filtered (0.01–0.08 Hz) to minimize the effects of low-frequency drift and high-frequency noise. Then, each brain region with altered ALFF was used as a region of interest (ROI) for seed-based dFC analysis (Li et al., 2019; Christiaen et al., 2020). The same sliding-window parameters used in the dALFF analysis were applied to derive windowed time series. Within each window, whole-brain FC maps for each ROI were computed by calculating Pearson correlation coefficients between the average time series of all voxels in the ROI and the time series of all other brain voxels. Fisher’s *r*-to-*z* transformation was applied to all FC maps to improve normality. The SD of the FC maps across time windows was calculated for each subject to characterize variations in individual ROI-to-whole-brain FC (Harlalka et al., 2019; Xue et al., 2022).

### Statistical analysis

Voxel-based comparisons of dALFF between the two groups were conducted using a general linear model, with gender and age as covariates. Cluster-level family-wise error (FWE) correction was applied to control for multiple comparisons with a voxel-level uncorrected threshold of *p* < 0.001 and a cluster-level corrected threshold of *p* < 0.05. The same statistical model and threshold were used for seed-based dFC analysis between the two groups.

To investigate the relationship between dALFF and dFC, a correlation analysis was carried out. This analysis aimed to explore how changes in spontaneous brain activity (as measured by dALFF) are related to alterations in functional interactions between regions (as measured by dFC). Understanding this relationship is crucial because it can provide insights into the underlying neural mechanisms and how different aspects of brain function are interconnected.

Additionally, the relationship between these neural alterations and the duration of *Pingju* training was explored through a correlation analysis to assess the association between the training duration and the observed changes in dALFF and dFC in the identified brain regions. This analysis aimed to determine whether longer training periods were linked to more significant neural changes. The significance level for these analyses was adjusted using the Bonferroni correction, with a threshold set at *p* < 0.05.

### Validation analysis

To ensure the robustness of our findings, we conducted a validation analysis using varying parameters for window length and step size to assess the consistency of our primary results. Specifically, we analyzed the data using four combinations: (1) a window length of 30 TRs with a step size of 1 TR, (2) a window length of 70 TRs with a step size of 1 TR, (3) a window length of 50 TRs with a step size of 5 TRs, and (4) a window length of 50 TRs with a step size of 10 TRs. We extracted the mean dALFF and dFC values from the significant ROIs identified in the primary results using these alternative parameters. Subsequently, a general linear model was applied to compare the dALFF and dFC values between groups, with gender and age included as covariates. Additionally, partial correlation analyses were performed to investigate the relationships both within dALFF and dFC and between these neural changes and the duration of training, controlling for age and sex.

## Results

### Demographics of the participants

One *Pingju* actor was excluded from the study due to excessive head motion (mean FD > 0.3 mm). The final sample consisted of 19 *Pingju* actors and 20 untrained subjects. Gender difference was assessed using a chi-square test, while age difference was evaluated with a two-sample *t*-test. The results showed that the two groups were well-matched in terms of gender (9 males in the *Pingju* actor group, 10 males in the untrained group, *p* = 0.98), handedness (all participants were right-handed), and age (30.47 ± 2.29 years for the *Pingju* actors, 29.10 ± 4.82 years for the untrained subjects, *p* = 0.27). Additionally, the *Pingju* actors had an average of 23.53 ± 1.43 years of training. The detailed demographic data are presented in Table 1.

**TABLE 1:** Demographic information of professional *Pingju* actors and untrained subjects.

Variables	Actors	Untrained subjects	*p*-value
Gender (male/female)	9/10	10/10	0.98
Age (years)	30.47 ± 2.29	29.10 ± 4.82	0.27
Handedness (right/left)	20/0	20/0	–
Years of training	23.53 ± 1.43	–	–

The *p* values were obtained by a two-sample *t*-test for age, and a chi-square test for gender. SD, standard deviation.

### Differences in dALFF and dFC

**FIGURE 1:**
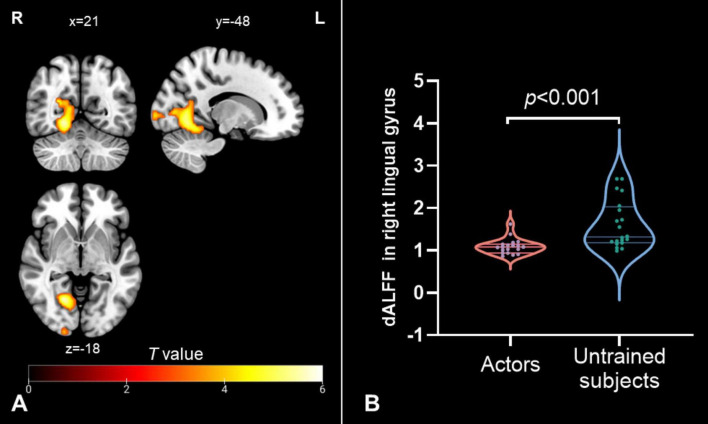
Dynamic ALFF changes in professional *Pingju* actors. **(A)** Brain regions showing significantly lower dALFF in professional *Pingju* actors compared to untrained subjects (cluster-level FWE *p* < 0.05), particularly in the right lingual gyrus (highlighted in orange-yellow). **(B)** Violin plots comparing the mean dALFF values in the right lingual gyrus between professional *Pingju* actors and untrained subject. Professional *Pingju* actors show significantly lower dALFF than untrained subjects (*p* < 0.001). Horizontal lines indicate median and quartiles. Abbreviations: dALFF, dynamic amplitude of low-frequency fluctuation; L, left; R, right.

**FIGURE 2:**
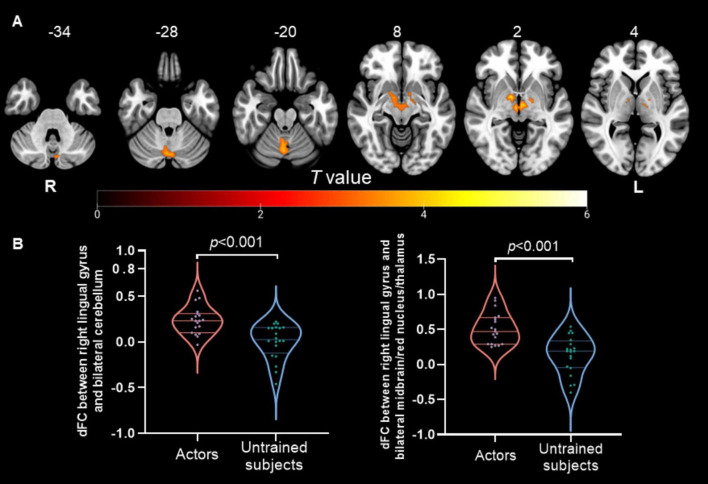
Dynamic FC changes in professional *Pingju* actors using the right lingual gyrus as the seed region. **(A)** Brain regions showing significantly increased dFC in professional *Pingju* actors compared to untrained subjects (cluster-level FWE *p* < 0.05). The regions include the bilateral cerebellum and bilateral midbrain/red nucleus/thalamus (highlighted in orange-yellow). **(B)** Violin plots comparing the mean dFC values between the right lingual gyrus and the bilateral cerebellum (left graph), and between the right lingual gyrus and the bilateral midbrain/red nucleus/thalamus (right graph). Professional *Pingju* actors show significantly higher dFC in both comparisons (*p* < 0.001). Horizontal lines indicate median and quartiles. Abbreviations: dFC, the dynamic functional connectivity; L, left; R, right.

The dALFF analysis revealed significant differences in spontaneous local brain activity between the two groups, with professional *Pingju* actors showing significantly lower dALFF in the right lingual gyrus compared to untrained individuals (*p* < 0.05, cluster-level FWE correction; Figure 1 and Table 2). For the subsequent dFC analysis, the right lingual gyrus was used as the seed region. The results indicated that professional *Pingju* actors showed increased dFC between the right lingual gyrus and several regions, including the bilateral cerebellum, and bilateral midbrain/red nucleus/thalamus, compared to untrained individuals (*p* < 0.05, cluster-level FWE correction; Figure 2 and Table 2).

**TABLE 2:** The brain regions with significant dALFF and dFC difference in professional *Pingju* actors.

Brain regions	Cluster Size (Voxels)	Peak MNI coordinate			Peak *T*
		*x*	*y*	*z*	
***dALFF alteration***					
Right lingual gyrus	347	21	−48	−18	4.39
***dFC alteration***					
Bilateral cerebellum	96	3	−72	−27	3.98
Bilateral midbrain/ red nucleus/thalamus	146	0	−15	−6	4.97

dALFF, the dynamic amplitude of low-frequency fluctuation; dFC, the dynamic functional connectivity; MNI, Montreal Neurological Institute; *T*, *t*-statistic.

### Correlation analysis

**FIGURE 3:**
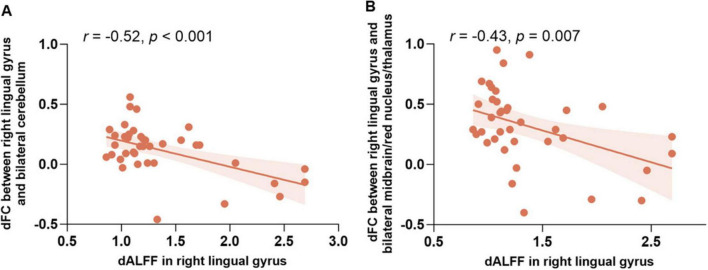
The relationship between dALFF and dFC in the right lingual gyrus. **(A)** Scatter plot showing the negative correlation between dALFF in the right lingual gyrus and dFC between the right lingual gyrus and the bilateral cerebellum (*r* = –0.52, *p* < 0.001). **(B)** Scatter plot showing the negative correlation between dALFF in the right lingual gyrus and dFC between the right lingual gyrus and the bilateral midbrain/red nucleus/thalamus (*r* = –0.43, *p* = 0.007). Abbreviations: dALFF, the dynamic amplitude of low-frequency fluctuation; dFC, the dynamic functional connectivity.

The correlation analysis examining the relationship between dALFF and dFC revealed significant findings. As shown in Figure 3, the dALFF of the right lingual gyrus was negatively correlated with the dFC between the right lingual gyrus and the cerebellum (*r* = −0.52, *p* < 0.001); similarly, it was also negatively correlated with the dFC between the right lingual gyrus and the bilateral midbrain/red nucleus/thalamus (*r* = −0.43, *p* = 0.007).

**FIGURE 4:**
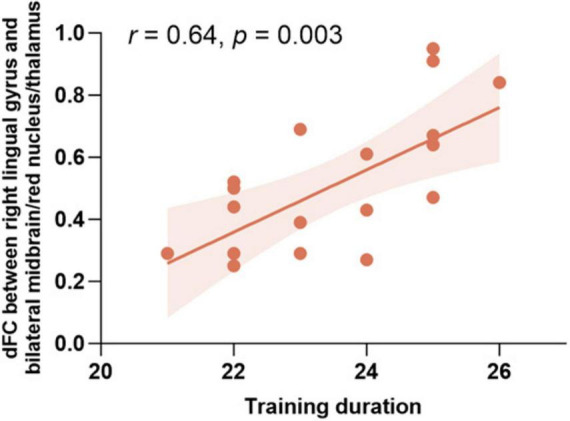
Correlation between training duration and dFC between the right lingual gyrus and the bilateral midbrain/red nucleus/thalamus. Scatter plot showing the positive correlation between the duration of *Pingju* training and dFC between the right lingual gyrus and the bilateral midbrain/red nucleus/thalamus (*r* = 0.64, *p* = 0.003). dFC, the dynamic functional connectivity.

Further analysis explored the relationships between these neural alterations and the duration of *Pingju* training. After applying the Bonferroni correction (*p* < 0.05/3 = 0.0167), a significant positive correlation was found between the dFC between the right lingual gyrus and the bilateral midbrain/red nucleus/thalamus and the duration of training (*r* = 0.64, *p* = 0.003; Figure 4). However, the dALFF of the right lingual gyrus and the dFC between the right lingual gyrus and bilateral cerebellum were not significantly correlated with the duration of training.

### Validation analysis

Our validation analysis demonstrated the consistency of our primary findings across a range of analytical conditions. When we examined dALFF and dFC values in significant ROIs, the results were robust across all testing configurations: (1) a window length of 30 TRs with a step size of 1 TR, (2) a window length of 70 TRs with a step size of 1 TR, (3) a window length of 50 TRs with a step size of 5 TRs, and (4) a window length of 50 TRs with a step size of 10 TRs. These varied settings confirmed that the significant differences initially observed in the standard setting of 50 TRs and 1 TR step size persisted. Additionally, the partial correlation analyses reinforced the relationships within dALFF and dFC and between these neural measures and the duration of *Pingju* training, remaining stable regardless of the analytical parameters used. For detailed results, please see Supplementary Figures 1–4.

## Discussion

To the best of our knowledge, this study represents the first attempt to investigate the effects of long-term *Pingju* training on dynamic brain activity and connectivity. We found that professional *Pingju* actors exhibited significantly lower dALFF in the right lingual gyrus compared to untrained individuals. Additionally, the actors showed increased dFC between the right lingual gyrus and both the bilateral cerebellum and the bilateral midbrain/red nucleus/thalamus. These findings suggest that extensive training in *Pingju* may induce neuroplastic changes by altering local brain activity and the FC between brain regions, highlighting the importance of understanding how specialized training in the arts can influence brain function.

Our study identified a significant decrease in the dALFF in the right lingual gyrus among professional *Pingju* actors compared with untrained individuals. The right lingual gyrus is predominantly associated with visual processing (Bogousslavsky et al., 1987; Palejwala et al., 2021; Chai et al., 2024), including the interpretation of complex visual stimuli such as shapes and spatial orientation (Hill et al., 2019; Baltaretu et al., 2021). In *Pingju*, performers must develop exceptional visual-motor coordination to execute precise movements and interpret visual cues on stage. This extensive training beginning at a young age may result in the enhanced specialization and efficiency of the neural circuits within the lingual gyrus. The observed decrease in dALFF may reflect a more streamlined neural processing mechanism in this region, as the brain adapts to the repetitive and intensive visual tasks required by *Pingju*. This reduction indicates that the actors’ brains have become more efficient at handling visual information, reducing the need for heightened spontaneous neural activity. Similar patterns of neural adaptation have been reported in studies on neuroplasticity related to musical training, where reduced mean diffusivity in the lingual gyrus was observed (Olszewska et al., 2021). The decrease in dALFF within the right lingual gyrus observed in our current study may, therefore, suggest a similar neuroplastic adaptation, optimizing the brain’s response to the specific demands of *Pingju* performance, such as visual-motor integration and spatial awareness.

The results of the dFC analysis revealed that professional *Pingju* actors exhibited increased dFC between the right lingual gyrus and bilateral cerebellum, as well as the bilateral midbrain/red nucleus/thalamus. This enhanced connectivity illustrates the intricate relationship between motor coordination, sensory processing, and cognitive functions (Higashionna et al., 2017; Pastor-Cerezuela et al., 2020), which is crucial for the highly specialized and demanding performance in *Pingju*. The cerebellum plays a pivotal role in motor control, balance, and coordination (Manto et al., 2012; Rudolph et al., 2023). Increased connectivity between the right lingual gyrus and the bilateral cerebellum suggests that *Pingju* training strengthens the integration of visual and motor functions. This enhancement likely supports the precise and fluid movements required on stage, allowing performers to execute complex sequences with greater accuracy and timing. Similar neuroplastic adaptations have been observed in other artistic professions, such as musicians and dancers, where extensive training also leads to enhanced FC between motor, sensory, and cognitive brain regions (Schlaug, 2001; Eierud et al., 2023). Additionally, the midbrain, red nucleus, and thalamus are involved in sensorimotor integration, motor coordination, and alertness (Yanaka et al., 2010; Basile et al., 2021; Wolff et al., 2021; Mao et al., 2022). The increased dFC between the right lingual gyrus and these regions indicates a more robust communication network supporting the sensory-motor demands of *Pingju*. The red nucleus, as part of the extrapyramidal system, is crucial for fine motor control (Ogut et al., 2023), and its enhanced connectivity with the right lingual gyrus may facilitate the intricate hand and body movements characteristic of *Pingju* performance. This finding aligns with research on ballet dancers, where enhanced connectivity in the extrapyramidal system has been linked to the refined motor skills required for complex dance routines (Burzynska et al., 2017). Overall, these results demonstrate how *Pingju* training enhances FC in brain regions involved in motor coordination and sensory integration, reflecting specialized neuroplastic adaptations that support the precise movements and complex performances required in this art form.

The correlation analysis revealed significant neural adaptations associated with long-term *Pingju* training. Specifically, the dALFF of the right lingual gyrus was negatively correlated with the dFC between the right lingual gyrus and both the cerebellum and the midbrain/red nucleus/thalamus. These findings suggest that lower spontaneous activity in the right lingual gyrus is linked to higher FC with these regions, indicating more efficient neural processing for the complex visual and motor tasks required in *Pingju*. Additionally, the duration of training positively correlates with the dFC between the right lingual gyrus and the midbrain/red nucleus/thalamus, highlighting enhanced connectivity with extended practice. These findings emphasize the adaptive changes in brain function due to specialized, long-term artistic training, enhancing neural efficiency and integration in regions critical for *Pingju* performance.

Several limitations of this study must be acknowledged. First, the sample size was relatively small, with only 20 professional *Pingju* actors and 20 untrained individuals. This limited sample size may affect the generalizability of the results to a broader population of opera performers or other individuals engaged in similar artistic training. Second, the cross-sectional design restricts our ability to draw causal conclusions about the effects of long-term *Pingju* training on brain function. Longitudinal studies are needed to better understand the causal relationships and the progression of neuroplastic changes over time. Finally, we did not collect data on several potential confounding factors, such as lifestyle differences, stress levels, or other forms of physical or cognitive training. These factors could independently influence brain activity and connectivity, potentially contributing to the observed differences in dALFF and dFC between *Pingju* actors and untrained subjects.

## Conclusion

In conclusion, our study demonstrated that long-term *Pingju* training induces significant neuroplastic changes, as evidenced by altered dALFF and dFC in the brain. Specifically, professional *Pingju* actors exhibited reduced dALFF in the right lingual gyrus, coupled with increased dFC between the right lingual gyrus and both the bilateral cerebellum and the bilateral midbrain/red nucleus/thalamus. These findings contribute to our understanding of how specialized, long-term artistic training can reshape brain function, reinforcing the potential of cultural practices like *Pingju* to modulate neural architecture. Future research should explore the cognitive and emotional implications of these neural changes, elucidating the impact of professional artistic training on brain health and cognitive functions.

## Data Availability

The original contributions presented in the study are included in the article/Supplementary material, further inquiries can be directed to the corresponding authors.
