# Supplementary material for: Neuroplastic changes induced by long-term Pingju training: insights from dynamic brain activity and connectivity
Source: Front Neurosci. 2024 Sep 27;18:1477181. doi: 10.3389/fnins.2024.1477181 (PMC11466935; doi:10.3389/fnins.2024.1477181)
Supplement: Supplementary file 1 [file Data_Sheet_1.docx]

**Supplementary Material**

**Supplementary Figures**


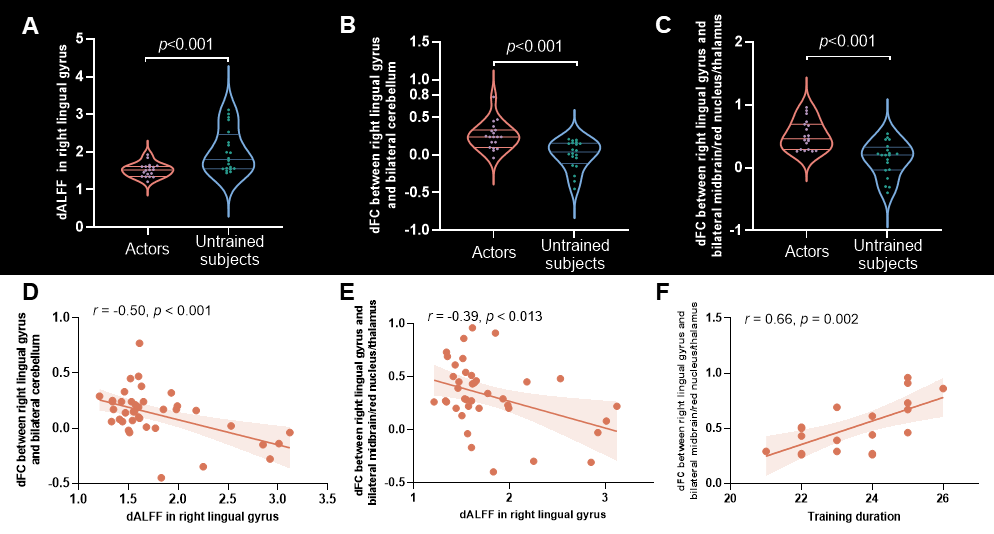


**Figure S1.** Validation analysis results in professional *Pingju* actors with a 30 TRs window length and a 1 TR step size. (A) Violin plot comparing the dALFF values in the right lingual gyrus between professional *Pingju* actors and untrained subjects. Horizontal lines indicate the median and quartiles. (B) Violin plot comparing the dFC values between the right lingual gyrus and the bilateral cerebellum. Horizontal lines indicate the median and quartiles. (C) Violin plot comparing the dFC values between the right lingual gyrus and the bilateral midbrain/red nucleus/thalamus. Horizontal lines indicate the median and quartiles. (D) Scatter plot showing a negative correlation between dALFF in the right lingual gyrus and dFC between the right lingual gyrus and the bilateral cerebellum. (E) Scatter plot showing a negative correlation between dALFF in the right lingual gyrus and dFC between the right lingual gyrus and the bilateral midbrain/red nucleus/thalamus. (F) Scatter plot showing a positive correlation between the duration of *Pingju* training and dFC between the right lingual gyrus and the bilateral midbrain/red nucleus/thalamus. Abbreviations: dALFF, dynamic amplitude of low-frequency fluctuation; dFC, dynamic functional connectivity.

**
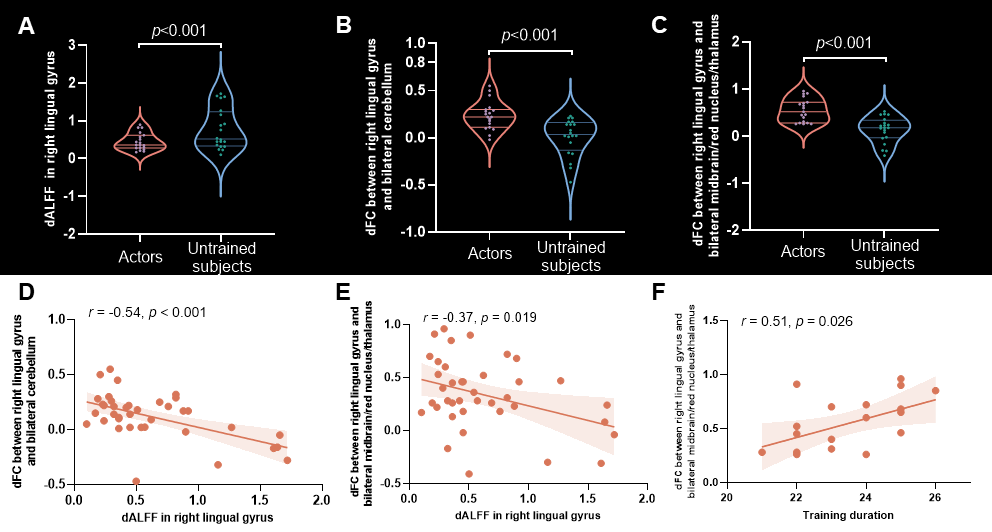
**

**Figure S2.** Validation analysis results in professional *Pingju* actors with a 70 TRs window length and a 1 TR step size. (A) Violin plot comparing the dALFF values in the right lingual gyrus between professional *Pingju* actors and untrained subjects. Horizontal lines indicate the median and quartiles. (B) Violin plot comparing the dFC values between the right lingual gyrus and the bilateral cerebellum. Horizontal lines indicate the median and quartiles. (C) Violin plot comparing the dFC values between the right lingual gyrus and the bilateral midbrain/red nucleus/thalamus. Horizontal lines indicate the median and quartiles. (D) Scatter plot showing a negative correlation between dALFF in the right lingual gyrus and dFC between the right lingual gyrus and the bilateral cerebellum. (E) Scatter plot showing a negative correlation between dALFF in the right lingual gyrus and dFC between the right lingual gyrus and the bilateral midbrain/red nucleus/thalamus. (F) Scatter plot showing a positive correlation between the duration of *Pingju* training and dFC between the right lingual gyrus and the bilateral midbrain/red nucleus/thalamus. Abbreviations: dALFF, dynamic amplitude of low-frequency fluctuation; dFC, dynamic functional connectivity.

**
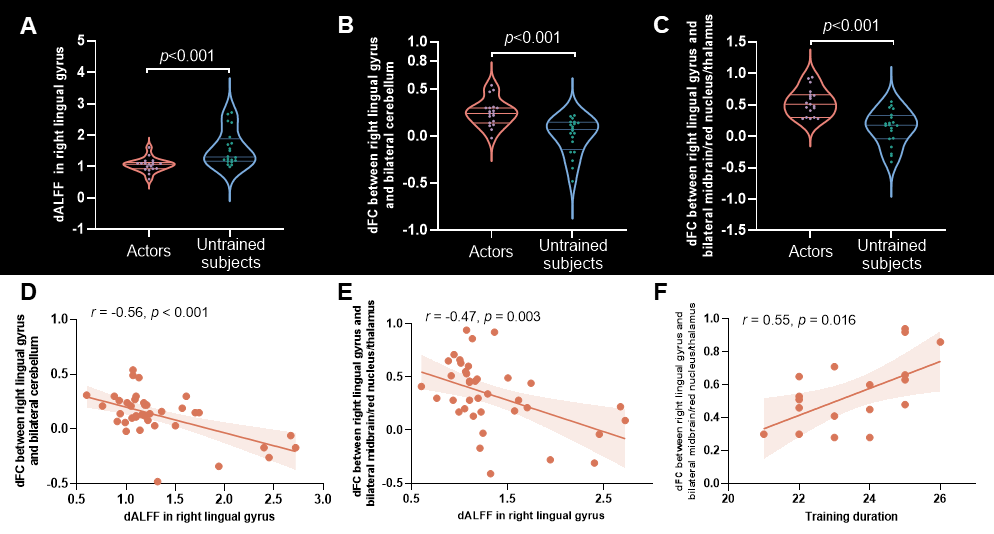
**

**Figure S3.** Validation analysis results in professional *Pingju* actors with a 50 TRs window length and a 5 TRs step size. (A) Violin plot comparing the dALFF values in the right lingual gyrus between professional *Pingju* actors and untrained subjects. Horizontal lines indicate the median and quartiles. (B) Violin plot comparing the dFC values between the right lingual gyrus and the bilateral cerebellum. Horizontal lines indicate the median and quartiles. (C) Violin plot comparing the dFC values between the right lingual gyrus and the bilateral midbrain/red nucleus/thalamus. Horizontal lines indicate the median and quartiles. (D) Scatter plot showing a negative correlation between dALFF in the right lingual gyrus and dFC between the right lingual gyrus and the bilateral cerebellum. (E) Scatter plot showing a negative correlation between dALFF in the right lingual gyrus and dFC between the right lingual gyrus and the bilateral midbrain/red nucleus/thalamus. (F) Scatter plot showing a positive correlation between the duration of *Pingju* training and dFC between the right lingual gyrus and the bilateral midbrain/red nucleus/thalamus. Abbreviations: dALFF, dynamic amplitude of low-frequency fluctuation; dFC, dynamic functional connectivity.

**
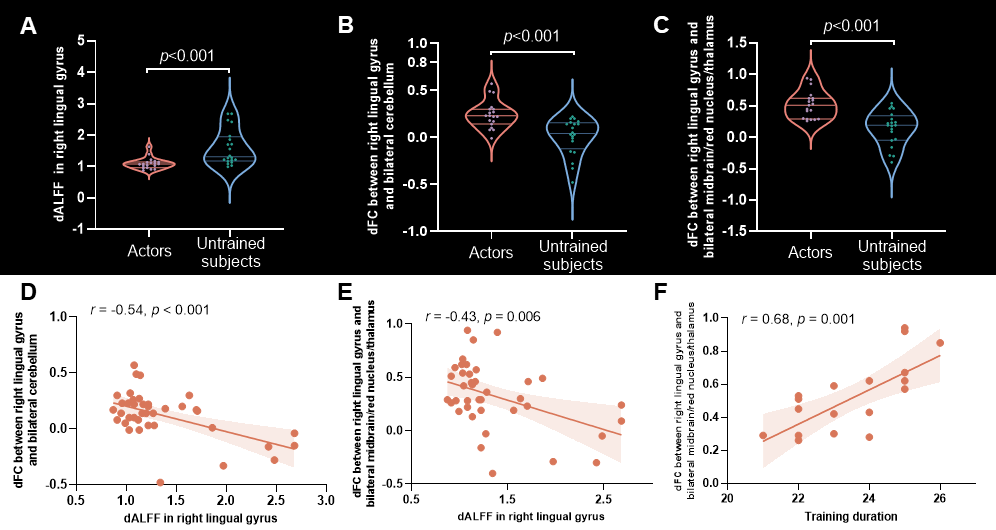
**

**Figure S4.** Validation analysis results in professional *Pingju* actors with a 50 TRs window length and a 10 TRs step size. (A) Violin plot comparing the dALFF values in the right lingual gyrus between professional *Pingju* actors and untrained subjects. Horizontal lines indicate the median and quartiles. (B) Violin plot comparing the dFC values between the right lingual gyrus and the bilateral cerebellum. Horizontal lines indicate the median and quartiles. (C) Violin plot comparing the dFC values between the right lingual gyrus and the bilateral midbrain/red nucleus/thalamus. Horizontal lines indicate the median and quartiles. (D) Scatter plot showing a negative correlation between dALFF in the right lingual gyrus and dFC between the right lingual gyrus and the bilateral cerebellum. (E) Scatter plot showing a negative correlation between dALFF in the right lingual gyrus and dFC between the right lingual gyrus and the bilateral midbrain/red nucleus/thalamus. (F) Scatter plot showing a positive correlation between the duration of *Pingju* training and dFC between the right lingual gyrus and the bilateral midbrain/red nucleus/thalamus. Abbreviations: dALFF, dynamic amplitude of low-frequency fluctuation; dFC, dynamic functional connectivity.
